# Endothelial Microparticle-Derived Reactive Oxygen Species: Role in Endothelial Signaling and Vascular Function

**DOI:** 10.1155/2016/5047954

**Published:** 2016-05-23

**Authors:** Dylan Burger, Maddison Turner, Mercedes N. Munkonda, Rhian M. Touyz

**Affiliations:** ^1^Kidney Research Centre, Ottawa Hospital Research Institute, University of Ottawa, 2501-451 Smyth Road, Ottawa, ON, Canada K1H 8M5; ^2^Institute of Cardiovascular & Medical Sciences, BHF Glasgow Cardiovascular Research Centre, University of Glasgow, 126 University Place, Glasgow G12 8TA, UK

## Abstract

Endothelial microparticles are effectors of endothelial damage; however mechanisms involved are unclear. We examined the effects of eMPs on cultured endothelial cells (ECs) and isolated vessels and investigated the role of eMP-derived reactive oxygen species (ROS) and redox signaling in these processes. eMPs were isolated from EC media and their ability to directly produce ROS was assessed by lucigenin and liquid chromatography. Nicotinamide adenine dinucleotide phosphate oxidase (Nox) subunits were probed by Western blot. ECs were treated with eMPs and effects on kinase signaling, superoxide anion (O_2_^∙−^) generation, and nitric oxide (NO) production were examined. Acetylcholine-mediated vasorelaxation was assessed by myography in eMP-treated mesenteric arteries. eMPs contained Nox1, Nox2, Nox4, p47^phox^, p67^phox^, and p22^phox^ and they produced ROS which was inhibited by the Nox inhibitor, apocynin. eMPs increased phosphorylation of ERK1/2 and Src, increased O_2_^∙−^ production, and decreased A23187-induced NO production in ECs. Pretreatment of eMPs with apocynin diminished eMP-mediated effects on ROS and NO production but had no effect on eMP-mediated kinase activation or impairment in vasorelaxation. Our findings identify a novel mechanism whereby eMP-derived ROS contributes to MP bioactivity. These interactions may be important in conditions associated with vascular injury and increased eMP formation.

## 1. Introduction

The endothelium plays a critical role in the regulation of blood flow, cellular trafficking, coagulation, and inflammation [1, 2]. Normal endothelial function requires a dynamic, controlled communication system between endothelial cells (ECs) and vascular smooth muscle cells, fibroblasts, and immune cells that employ electrical signals, cell-cell/cell-matrix contacts, cytokines/proteins, small molecules, and gases such as nitric oxide [2–4]. In addition, intercellular communication may involve the release of extracellular vesicles, which can function as intercellular carriers of ligands, enzymes, RNA, and miRNA [5–7]. Vascular cells have been shown to release vesicles of differing origins that contribute to many (patho)physiological processes including angiogenesis, coagulation, inflammation, and fibrosis [8].

Microparticles (MPs, sometimes referred to as microvesicles or ectosomes) are 100–1000 nm anuclear vesicles formed following cytoskeletal and membrane reorganization and release from cells into the extracellular milieu [7, 8]. In biological samples, alterations in MP levels may be indicative of underlying pathology, correlate with measures of vascular dysfunction, and predict risk of adverse cardiovascular events [8–10]. MPs are also potent autocrine/paracrine signals for various cellular responses [11]. For example, endothelial MPs promote cell senescence, oxidative stress, coagulation, inflammation, and apoptosis [8, 12–15]. Amongst the many responses initiated by endothelial MPs in ECs is the induction of reactive oxygen species (ROS) production. Initial studies by Brodsky et al. showed that endothelial MPs induce O_2_^∙−^ production in cultured rat renal microvascular ECs and in* ex vivo* aortic rings [16]. A follow-up study implicated ROS in the antiangiogenic effects of endothelial MPs [17]. Similarly ROS production has been shown in human umbilical vein ECs exposed to endothelial MPs [18–20]. We have observed that exogenous endothelial MPs induce ROS production in ECs through both mitochondrial and nicotinamide adenine dinucleotide phosphate (NADPH) oxidases [12, 13]. More controversially, it has been suggested that endothelial MPs may directly produce ROS. Brodsky et al. examined* de novo* ROS production by endothelial MPs by DHE staining and observed p22^phox^ within endothelial MPs [16]. NADPH oxidase activity has also been reported in aortic endothelial MPs and may be influenced by glucose exposure [21]. However, to date, there has been no direct evidence that MPs possess the complete machinery necessary to produce ROS, nor has the role of* de novo* ROS production on MP-mediated EC responses been evaluated.

The purpose of this study was to evaluate whether endothelial MPs produce ROS and to evaluate the potential regulatory role of MP-derived ROS on EC signaling and vascular function.

## 2. Materials and Methods

### 2.1. Reagents

NaCl, D-glucose, KH_2_PO_4_, NaF, dihydroethidium (DHE, stock 10 mM in DMSO), Tris-base, Dulbecco's Modified Eagle Media (DMEM), fetal calf serum (FCS), penicillin/streptomycin, 1x minimal essential amino acids, and 4-amino-5-methylamino-2′,7′-difluorofluorescein diacetate (DAF, stock 10 mM in DMSO) were all purchased from Thermo Fisher Scientific (Waltham, MA, USA). Acetylcholine, phenylephrine, leupeptin, aprotinin, sodium orthovanadate, pepstatin, phenylmethylsulfonyl fluoride (PMSF), NaHCO_3_, ethylene glycol tetraacetic acid (EGTA), A23187 (calcium ionophore, stock 10 mM in DMSO), sodium deoxycholate, NP40, lucigenin, diethylenetriaminepentaacetic acid (DTPA), apocynin, and endothelial cell growth supplement were all purchased from Sigma-Aldrich (St. Louis, MO, USA). KCl, CaCl_2_, MgSO_4_, and ethylene-diaminetetraacetic acid (EDTA) were all purchased from EMD Millipore (Billerica, MA, USA). Sodium dodecyl sulfate (SDS) and NADPH were purchased from VWR (Radnor, PA, USA). Alexa-647-labeled Annexin V were purchased from BioLegend (San Diego, CA, USA). Heparin was purchased through Leo Pharma (Thornhill, ON, Canada). A complete list of all antibodies used and their sources is provided in Table 1.

### 2.2. Animals

The study was approved by the Animal Ethics Committee of the University of Ottawa and performed according to the recommendations of the Canadian Council for Animal Care.

### 2.3. Cell Culture

ECs were isolated from aortas of C57BL6/J mice as we previously described [13, 22]. Cells were cultured on attachment factor-coated polystyrene dishes in DMEM containing 10% FCS, 50 mg/L of EC growth supplement, 10 U/mL heparin, 100 U/mL penicillin/streptomycin, and 1x minimal essential amino acids and placed in a humidified incubator at 37°C and 5% CO_2_.

### 2.4. Endothelial Microparticle Isolation and Quantification

Endothelial MPs were isolated from media collected from EC cultures using a modification of our previously described methods [13, 23]. Samples were centrifuged at 2500 g for 10 minutes at 4°C to obtain cell-free media. MPs were then pelleted from cell-free media by centrifugation at 20000 ×g for 20 minutes at 4°C. Endothelial MPs were confirmed as such and quantified by flow cytometry using an Alexa-647-labeled Annexin V (0.5 *μ*g/mL final concentration in Annexin V binding buffer) to identify events as MPs. As a negative control, a subpopulation of MPs were resuspended in Annexin V binding buffer lacking calcium, which is necessary for Annexin V binding to phosphatidylserine. MPs were centrifuged and resuspended in assay-specific buffers (indicated below) or 1x phosphate buffered saline for cell treatment studies. A final concentration of 10^5^ MPs/mL, previously identified to exert effects on cultured ECs, was used for treatments [12, 13].

### 2.5. Measurement of ROS Production

ROS production was measured by lucigenin chemiluminescence and by DHE/HPLC in MPs and ECs. For chemiluminescence, MPs were isolated from 100 mL of media and lysed in a buffer containing (20 mM of KH_2_PO_4_, 1 mM of EGTA, 1 *μ*g/mL of aprotinin, 1 *μ*g/mL of leupeptin, 1 *μ*g/mL of pepstatin, and 1 mM of PMSF). Following lysis, 50 *μ*L of sample was added to 175 *μ*L of assay buffer (50 mM of KH_2_PO_4_, 1 mM of EGTA, and 150 mM of sucrose) and 5 *μ*M lucigenin. NADPH (1 mM) was added to the suspension (300 *μ*L) containing lucigenin. Luminescence was measured on a Berthold Orion II luminometer (Berthold Detection Systems, Pforzheim, Germany) in 1 s readings over a total of 30 cycles before and after stimulation with NADPH. A buffer blank was subtracted from each reading. As a negative control, the high-speed supernatant obtained during MP isolation was also assessed. The results are expressed as counts per mg of protein.

For HPLC studies, MPs were isolated from 100 mL of media and incubated in DHE (50 *μ*M final concentration in a buffer containing 1.3 mM CaCl_2_, 0.8 mM MgSO_4_, 5.4 mM KCl, 0.4 mM KH_2_PO_4_, 4.3 mM NaHCO_3_, 137 mM NaCl, 0.3 mM Na_2_HPO_4_, 5.6 mM glucose, and 100 *μ*M DTPA, pH 7.4) for 30 minutes in the presence and absence of the Nox inhibitor, apocynin (10 *μ*M). DHE oxidation was then assessed by HPLC as described previously [13]. As a negative control, the high-speed supernatant obtained during MP isolation was also assessed.

### 2.6. Western Blot Analysis

Western blotting was used to examine the presence of NADPH oxidase subunits in endothelial MPs and to assess intracellular signaling responses in cultured ECs. Membranes were probed with anti-phosphorylated extracellular signal-related kinase (ERK1/2), anti-total ERK1/2, anti-phosphorylated Src, anti-total Src, anti-phosphorylated Akt, anti-total Akt, anti-phosphorylated (ser1177) eNOS, anti-phosphorylated (thr-495) eNOS, anti-total eNOS, anti-Nox1, anti-Nox2, anti-Nox4, anti-p22^phox^, anti-p47^phox^, and anti-p67^phox^. Membranes were then washed in TBS-T and incubated with horse-radish peroxidase- (HRP-) conjugated secondary antibodies in milk for 1 h. Membranes were probed for immunoreactivity by chemiluminescence. Quantification of blots was performed by densitometry (Image J, National Institute of Health, Baltimore, MD, USA).

### 2.7. Nitric Oxide Production

Measurement of EC nitric oxide production was performed using a fluorescence-based assay with live cell imaging [24–26]. Cells were loaded with the fluorescent NO-sensitive dye DAF (0.5 *μ*M final concentration) and incubated at 37°C for 30 minutes. Coverslips containing DAF-loaded cells were placed in a temperature regulated (37°C) chamber mounted on the stage of an inverted microscope in Krebs-Ringer phosphate (KRP) buffer (120 mM NaCl, 4.8 mM KCl, 0.54 mM CaCl_2_, 1.2 mM MgSO_4_, 11 mM glucose, and 15.9 mM sodium phosphate, pH 7.2). Changes in cellular DAF fluorescence intensities were simultaneously acquired for multiple cells (defined regions of interest) using a Stallion Digital Hi-Speed Multi-Channel Imaging System (Zeiss, Germany) with 495 nm excitation and 515 nm emission. Changes in NO induced by exposure to the calcium ionophore A23187 (1 *μ*M final concentration) were measured in relation to baseline fluorescence as described elsewhere [24–26]. Unstimulated and A23187 stimulated NO production was observed as the difference between the NO content measured in the presence (total) and absence (basal) of treatment and expressed as percentage of the basal considered as 100%.

### 2.8. Myography

Second-order branches of mouse mesenteric artery were isolated and cleaned of adhering fat and connective tissue. Vessels segments (1.5–2 mm) were mounted on a wire myograph and equilibrated in a physiological saline solution (in mM; 130 NaCl, 14.9 NaHCO_3_, 4.7 KCl, 1.18 KH_2_PO_4_, 1.17 MgSO_4_, 1.6 CaCl_2_, 0.026 EDTA, and 5.5 glucose, pH 7.4, 37°C) which was bubbled continuously with 95% O_2_ and 5% CO_2_. Endothelium-dependent relaxation was assessed in the presence and absence of endothelial MPs (10^5^ MPs/mL) as previously described [27]. Vessels were preincubated with MPs for 30 minutes. Endothelium-dependent relaxation was then examined by measuring dilatory responses to acetylcholine (1 nM–10 *μ*M) in vessels precontracted with phenylephrine at a concentration which was found to cause approximately 80% maximal contraction.

### 2.9. Statistical Analysis

Results are expressed as mean ± SEM and were analyzed using a one-way ANOVA with Bonferroni's posttest using Prism 5.0 (GraphPad Software, Inc., La Jolla, CA, USA). *P* < 0.05 was considered statistically significant.

## 3. Results

### 3.1. MPs Possess NADPH Oxidases and Generate ROS

**Figure 1:**
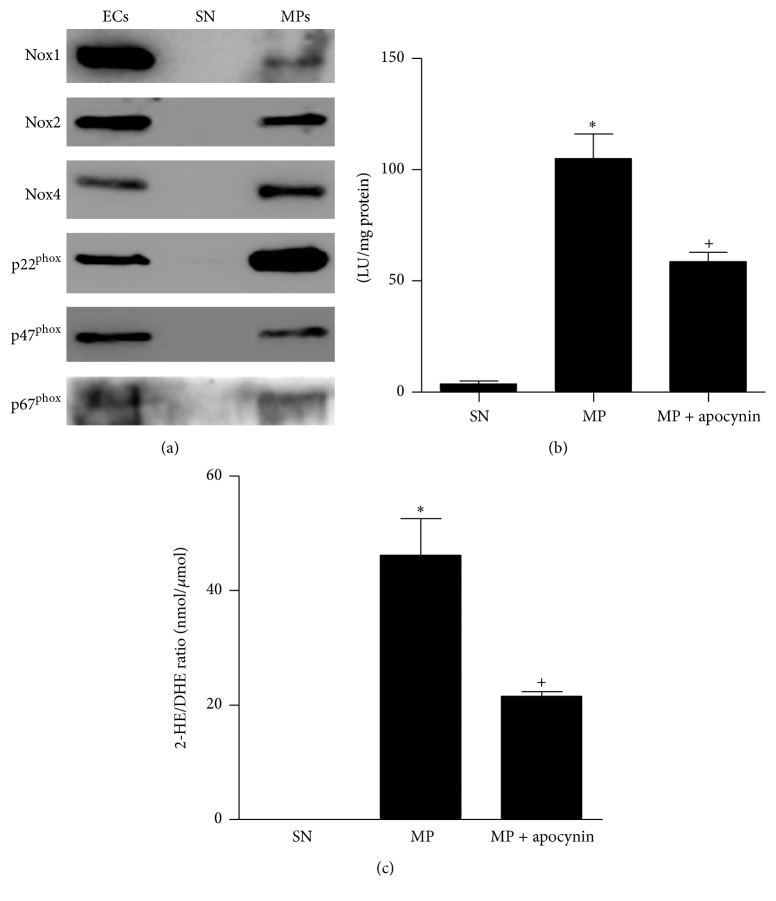
Nox machinery and ROS production in endothelial cells (EC), supernatant (SN), and endothelial microparticles (MP). (a) Expression of Nox1, Nox2, Nox4, p22^phox^, p47^phox^, and p67^phox^ was measured by Western blot analysis in EC, SN, and MP. MPs were isolated from the media of mouse aortic ECs. Blots are representative of at least four independent experiments. (b, c) ROS production by endothelial MPs as determined by lucigenin (b) and DHE/HPLC (c) in the presence and absence of the NADPH oxidase inhibitor apocynin (10 *μ*M). ^*∗*^ *P* < 0.01, MP versus SN, ^+^ *P* < 0.01 MP + apocynin versus MP, *n* = 3–5.

To determine whether MPs contain ROS-generating enzymes, we examined the presence of NADPH oxidase subunits in endothelial MPs. Both cultured ECs and endothelial MPs were found to contain Nox1, Nox2, Nox4, p22^phox^, p47^phox^, and p67^phox^ (Figure 1(a)). Compared with ECs, endothelial MPs were enriched in Nox4 and p22^phox^, while Nox1 and p67^phox^ were found at lower levels. No NADPH oxidase subunits were detectable in the high-speed supernatant obtained during MP isolation.

As shown in Figure 1(b) lucigenin chemiluminescence revealed that endothelial MPs produced O_2_^∙−^, a process that was attenuated by treatment with the NADPH oxidase inhibitor apocynin (5 *μ*M). Similarly, O_2_^∙−^ production was detectable by DHE/HPLC and was inhibited by apocynin (Figure 1(c)).

### 3.2. Effects of Endothelial MPs on Intracellular Signaling in Cultured ECs

**Figure 2:**
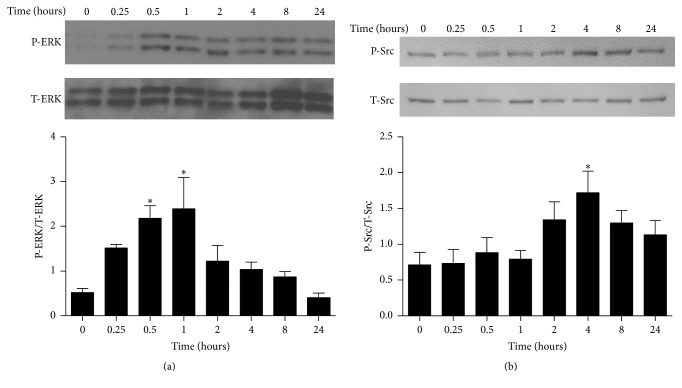
Effects of endothelial MPs on kinase signaling in cultured mouse aortic ECs. ECs were exposed to endothelial MPs (10^5^/mL) and phosphorylation of ERK1/2 (a) and Src (b) was examined by Western blot analysis. ^*∗*^ *P* < 0.05 versus no treatment (0 hours), *n* = 4–6.

In order to assess EC signaling following endothelial MP exposure, ECs were treated with endothelial MPs for 0–24 hours and phosphorylation of ERK1/2, Src, p38, and Akt kinases was measured by Western blot analysis. As shown in Figure 2. MPs increased ERK1/2 phosphorylation at 30 minutes and 1 hour while Src was increased at 4 hours. Conversely, Akt and p38 phosphorylation were unchanged over 24 hours (not shown).

### 3.3. Effects of Endothelial MPs on eNOS Activation and NO Production in Cultured ECs

**Figure 3:**
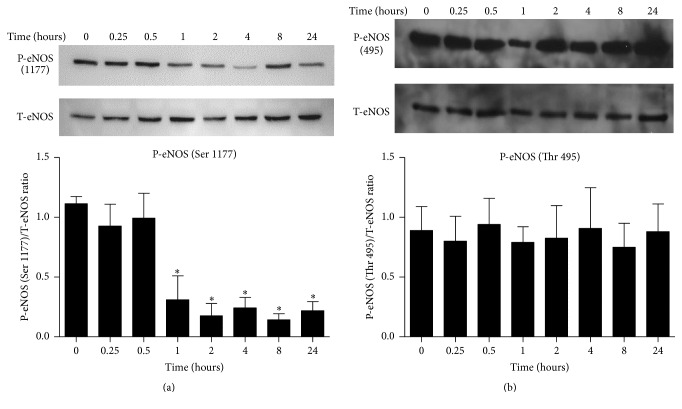
Effects of endothelial MPs on phosphorylation of eNOS in cultured mouse aortic ECs. ECs were exposed to endothelial MPs (10^5^/mL) and phosphorylation of eNOS at activation site, residue serine 1177 (a), and inhibitory site, residue threonine 495 (b), was examined by Western blot analysis. ^*∗*^ *P* < 0.05 versus no treatment (0 hours), *n* = 4–6.

**Figure 4:**
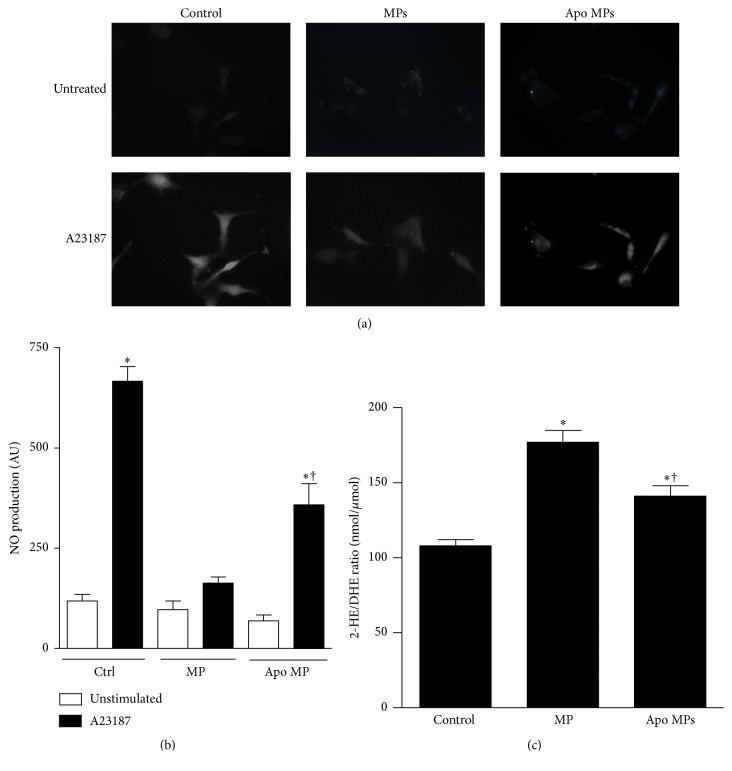
Effects of endothelial MPs on NO production (a, b) and ROS production (c) in cultured mouse aortic ECs. ECs were exposed to endothelial MPs (10^5^/mL, 4 hours) or endothelial MPs pretreated with the NADPH oxidase inhibitor apocynin (Apo MPs, 10^5^/mL, 4 hours). (a, b) NO production was measured in A23187 (10 *μ*M) stimulated ECs using diaminofluorescein diacetate and fluorescence microscopy. MP treatment impaired A23187 stimulated NO production and this process was partially attenuated by NADPH oxidase inhibition in microparticles. ^*∗*^ *P* < 0.05 versus unstimulated cells, ^†^ *P* < 0.05 versus stimulated control group, *n* = 11-12. (c) EC ROS production was assessed by DHE-HPLC. ^*∗*^ *P* < 0.05 versus unstimulated cells, ^†^ *P* < 0.05 versus stimulated control group, *n* = 3-4.

Stimulation of ECs with endothelial MPs was associated with an impairment in eNOS activation and NO production. Following exposure to endothelial MPs, phosphorylation of eNOS (serine 1177, associated with enzyme activation) was decreased suggesting impaired NO production (Figure 3(a)), while phosphorylation at threonine 495 (associated with enzyme inhibition) was unchanged (Figure 3(b)). Consistent with a decrease in eNOS activity, we observed a decrease in calcium-dependent NO production assessed by DAF-FM. Exposure to A23187 (10 *μ*M) was associated with a 5-6-fold increase in fluorescence, indicative of NO production, and consistent with previous reports [28] (Figure 4). Preincubation of ECs with endothelial MPs significantly impaired A23187-induced NO production in ECs.

### 3.4. Effects of MP-Derived ROS on Endothelial Cell Signaling

**Figure 5:**
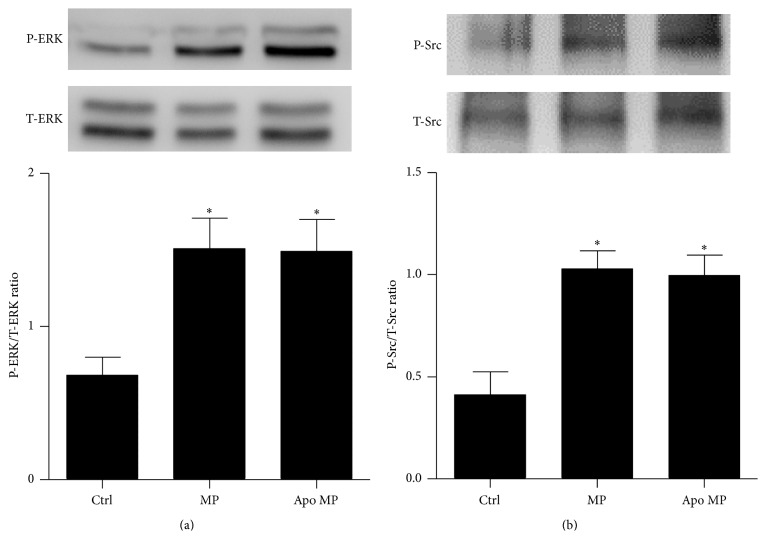
Effects of inhibition of NADPH oxidase activity on MP-mediated kinase signaling responses in mouse aortic ECs. Inhibition of NADPH oxidase with apocynin inhibition had no effect on endothelial MP-mediated activation of ERK1/2 (a) or Src (b). ^*∗*^ *P* < 0.05 versus control, *n* = 4.

To determine if* de novo* production of ROS plays a role in the biological actions of endothelial MPs endothelial MPs were pretreated with apocynin and administered to cultured ECs. Apocynin pretreatment partially attenuated endothelial MP-mediated inhibition of NO production (Figures 4(a) and 4(b)) and MP-mediated induction of ROS production (Figure 4(c)) but had no effect on endothelial MP-mediated ERK1/2 or Src phosphorylation (Figure 5).

### 3.5. Effects of Endothelial MPs on Endothelium-Dependent Vasorelaxation in Isolated Mesenteric Arteries

**Figure 6:**
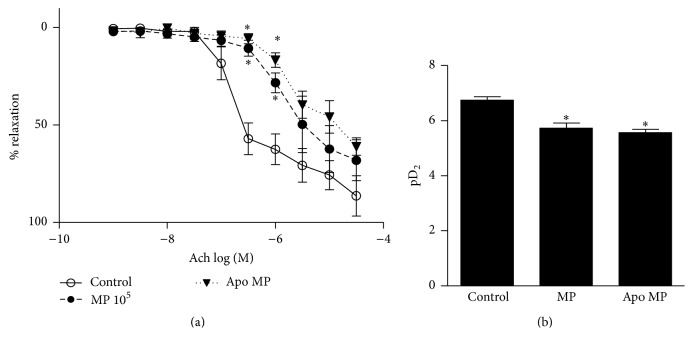
Effect of endothelial MPs on endothelium-dependent vasorelaxation in isolated mesenteric arteries. (a) Concentration-response curve to acetylcholine in untreated control vessels, those incubated with endothelial MPs (10^5^/mL, 30 minutes) or those incubated with endothelial MPs pretreated with the NADPH oxidase inhibitor apocynin (Apo MPs, 10^5^/mL, 30 minutes). ^*∗*^ *P* < 0.05 versus control vessels at corresponding dose, *n* = 3–8. (b) Sensitivity to acetylcholine (expressed as pD2 [−log⁡EC50]). ^*∗*^ *P* < 0.05 versus control, *n* = 3–8.

Finally, to determine whether endothelial MPs influence endothelium-dependent vasorelaxation we assessed endothelium-dependent relaxation induced by acetylcholine (ACh) in phenylephrine precontracted arteries (Figure 6). As shown in Figure 6, endothelium-dependent relaxation induced by ACh was impaired in mesenteric arteries pretreated with endothelial MPs. Preincubation of endothelial MPs with apocynin did not alter MP-mediated effects on mesenteric artery relaxation.

## 4. Discussion

Endothelial MPs influence EC function through redox-sensitive processes; however the molecular mechanisms underlying this are unclear. In the present study we show that endothelial MPs contain NADPH oxidase subunits, produce ROS in an NADPH oxidase-dependent fashion, and induce downstream effects on EC signaling. At the functional level, endothelial MPs influence vascular tone by inhibiting endothelium-dependent vasorelaxation. EC regulation by MPs occurs in a paracrine manner, where MP-derived ROS downregulates eNOS activity and stimulates ROS production, without influencing MAP kinases or Src. Our findings highlight novel signaling mechanisms through* de novo* ROS production by MPs and indicate that MP-derived ROS differentially regulate redox and kinase signaling in ECs. Such processes may influence endothelial function in conditions associated with increased endothelial MP production, such as in atherosclerosis, hypertension, or diabetes.

Microparticles typically possess membrane and cytoplasmic proteins/content of the parent cell from which they are derived. Here we show, similar to what has been demonstrated in ECs, that MPs express Nox isoforms and NADPH oxidase subunits, which are functionally active since ROS is produced in an apocynin-inhibitable manner. H_2_O_2_, a direct or indirect product of NADPH oxidase activation, is cell membrane-permeable and, as such, MP-derived ROS may influence ECs without direct physical contact. This observation may be of particular relevance in the* in vivo* setting where physical interaction between circulating MPs and the endothelium may be limited due to the presence of a glycocalyx, flow, or possibly atherosclerotic lesions. MPs also influence ECs through direct communication. We and others have reported that surface interactions involving ligand-mediated receptor activation contribute to endothelial MP bioactivity [13, 15, 29] while uptake of endothelial MPs has been implicated in processes associated with coagulation and inflammation [18, 30].

Although there is increasing interest in the role of endothelial MPs as redox biovectors of endothelial dysfunction and vascular inflammation, there is a paucity of information as to whether MPs themselves can generate ROS. Brodsky et al. reported that ROS are produced in isolated MPs and identified the presence of p22^phox^ [16]. However that study failed to identify the Nox machinery responsible for ROS production and in fact did not demonstrate Nox-dependence of the observed ROS production. Similarly, Jansen and colleagues reported ROS production by endothelial MPs and suggested that this may be dependent upon NADPH oxidase but the enzymatic machinery required for ROS production was again unclear [21]. Others have reported the presence of certain Nox machinery (p22^phox^, Nox2) in platelet-derived exosomes [31]. Our data show that endothelial MPs possess Nox1, Nox2, Nox4, p22^phox^, p47^phox^, and p67^phox^. Accordingly, ROS production in MPs may be mediated through multiple Nox isoforms. Of note is the observation that Nox1 and p67^phox^ were present at lower levels than seen in ECs while Nox4 and p22^phox^ were enriched in MPs.

We measured ROS production by two methods, lucigenin chemiluminescence and DHE/HPLC. Lucigenin assesses ROS production in lysed samples supplemented with NADPH such that endogenous sources of electrons are not required. On the other hand, DHE/HPLC methodology requires an intact system with an endogenous source of electrons for enzymatic activity. Thus it appears that endothelial MPs contain at least some source of NADPH that fuels ROS production. This observation is consistent with that reported by others where ROS was produced in MPs in the absence of NADPH supplementation [16, 21]. The source of electrons in endothelial MPs is presently unclear since MPs have not been shown to contain NADPH-generating enzymes. One possibility may be that NADPH in MPs is a residual component that derives from the cytosolic material of parent ECs released during MP formation.

Using apocynin, a pharmacological NADPH oxidase inhibitor, we showed that endothelial MPs require* de novo* ROS production for inhibition of eNOS-derived NO production and for endothelial MP-mediated induction of ROS production in ECs. While endothelial MPs reduced eNOS phosphorylation and NO production, we did not see any alterations in Akt signaling and mechanisms responsible for impaired NO production are unclear at this time. It is possible that endothelial MPs interfere with intracellular calcium handling or reduce eNOS cofactors. Alternatively, endothelial MPs may influence levels of protein phosphatase 2a (PP2A) which dephosphorylates eNOS at serine 1177 [32]. A recent proteomic analysis found PP2A present in human endothelial cell-derived MPs and it is conceivable that eMPs may transfer bioactive PP2A to target cells but this has not been shown experimentally.

Endothelial MP-mediated effects on intracellular ERK1/2 and Src as well as effects on vasorelaxation were not altered in apocynin-treated MPs suggesting that these processes are not influenced by MP-derived ROS. Such findings highlight the pleiotropic nature of MPs with both ROS-dependent and ROS-independent pathways contributing to endothelial MP-mediated signaling. Accordingly it is our belief that the observed ROS effects are not generalized phenomena but highly regulated processes. Exact mechanisms whereby MPs mediate their ROS-independent effects are unclear. One possibility is the activation of cell surface receptors by endothelial MPs. For example, we have previously reported that inhibition of epidermal growth factor receptor (an upstream activator of ERK1/2 and Src) blocks endothelial MP-mediated responses in cultured mouse aortic endothelial cells [13]. Endothelial MPs may conceivably activate other cell surface receptors; however this remains an area in need of investigation.

In summary, we demonstrate that endothelial MPs produce ROS through Nox-dependent processes, which contribute to endothelial O_2_^−^ generation and downregulation of NO generation without effect on ERK1/2 and Src activation or on MP-mediated impairment of vasorelaxation. Our findings indicate that MPs differentially influence EC function through redox-sensitive and insensitive processes. Modulation of vascular function by MPs appears to be NADPH oxidase/ROS-independent and may involve ERK1/2 and Src or other mechanisms. Cross talk between MPs and ECs may be important in conditions associated with vascular injury and increased endothelial MP formation.

## Figures and Tables

**Table 1:** List of antibodies used.

Antibody	Species	Supplier	Catalog number	Dilution factor
Nox1	Goat	Santa Cruz Biotechnology	sc-5821	1 : 2000
Nox2/gp91^phox^	Goat	Santa Cruz Biotechnology	sc-5827	1 : 1000
Nox4	Goat	Santa Cruz Biotechnology	sc-21860	1 : 1000
p22^phox^	Rabbit	Santa Cruz Biotechnology	sc-20781	1 : 1000
p47^phox^	Goat	Santa Cruz Biotechnology	sc-7660	1 : 1000
p67^phox^	Rabbit	Abcam	ab109366	1 : 1000
Phospho-ERK1/2 (Thr202/Tyr204)	Rabbit	Cell Signaling Technology	9101S	1 : 2000
Total ERK1/2	Rabbit	Cell Signaling Technology	9102S	1 : 2000
Phospho-Src (Tyr416)	Rabbit	Cell Signaling Technology	2101S	1 : 2000
Total Src	Rabbit	Cell Signaling Technology	2108	1 : 2000
Phospho-eNOS (Ser1177)	Rabbit	Cell Signaling Technology	9571	1 : 1000
Phospho-eNOS (Thr495)	Rabbit	Cell Signaling Technology	9574	1 : 1000
Total eNOS	Rabbit	Cell Signaling Technology	9572	1 : 2000
Phospho-AKT (Ser473)	Rabbit	Cell Signaling Technology	9271	1 : 2000
Total AKT	Rabbit	Cell Signaling Technology	9272	1 : 2000
Donkey anti-goat HRP	Donkey	Santa Cruz Biotechnology	sc-2033	1 : 2000
Goat anti-rabbit HRP	Goat	Santa Cruz Biotechnology	sc-2030	1 : 2000
Goat anti-mouse	Goat	Santa Cruz Biotechnology	sc-2031	1 : 2000
